# OUTDOOR PHYSICAL ACTIVITY AMONG OLDER ADULTS DURING THE COVID-19 PANDEMIC

**DOI:** 10.1093/geroni/igac059.064

**Published:** 2022-12-20

**Authors:** Justine Sefcik, Amina Panicker, Danielle Katsev, Annalisa Na, Sherry Greenberg, Rose Ann DiMaria-Ghalili

**Affiliations:** Drexel University, Philadelphia, Pennsylvania, United States; Drexel Univeresity, Philadelphia, Pennsylvania, United States; Drexel Univeresity, Philadelphia, Pennsylvania, United States; Drexel University, Philadelphia, Pennsylvania, United States; Monmouth University, Marjorie K. Unterberg School of Nursing & Health Studies, West Long Branch, New Jersey, United States; Drexel University, College of Nursing and Health Professions, Philadelphia, Pennsylvania, United States

## Abstract

There is a gap in understanding how the current pandemic is affecting older adults’ outdoor physical activity. This study aimed to explore older adults’ perceptions of their outdoor physical activity during the current pandemic. A qualitative descriptive approach was taken with a conventional content analysis. Participants were primarily recruited through ResearchMatch. Eighteen community-dwelling older adults were individually interviewed from geographical locations across the United States; 61.1% female, 88.9% White, mean age 76.4 (range 68-92), 5 ambulated with a cane or walker. We identified an overarching theme of Benefits and Motivation in which older adults conveyed wanting to maintain and improve their health and used the outdoors to continue physical activity since indoor activities decreased during the pandemic. Walking was expressed as the most frequent outdoor physical activity. Implications of these findings will be discussed which include supporting community improvements to facilitate older adults’ ease of maintaining a walking routine.

